# Perception of the Regulatory Change for Zolpidem Prescription by French General Practitioners and Its Relation to Prescription Behavior

**DOI:** 10.3390/jcm11082176

**Published:** 2022-04-13

**Authors:** Edouard-Jules Laforgue, Marion Istvan, Benoit Schreck, Marie Mainguy, Pascale Jolliet, Marie Grall-Bronnec, Caroline Victorri-Vigneau

**Affiliations:** 1 Nantes Université, CHU Nantes, Service de Pharmacologie Clinique—Centre d’Évaluation et d’Information sur la Pharmacovigilance-Addictovigilance, F-44000 Nantes, France; marion.istvan@chu-nantes.fr (M.I.); marie.mgy35@gmail.com (M.M.); pascale.jolliet@univ-nantes.fr (P.J.); caroline.vigneau@chu-nantes.fr (C.V.-V.); 2 Nantes Université, Univ Tours, CHU Nantes, CHU Tours, INSERM, MethodS in Patients-Centered Outcomes and HEalth Research, SPHERE, F-44000 Nantes, France; benoit.schreck@chu-nantes.fr (B.S.); marie.bronnec@chu-nantes.fr (M.G.-B.); 3 Nantes Université, CHU Nantes, Service d’Addictologie et de Psychiatrie de Liaison, F-44000 Nantes, France

**Keywords:** zolpidem, drug regulation, perception, general practitioner, prescription behavior

## Abstract

Background: To “limit the risk of abuse and misuse” and “encourage correct usage”, the French drug regulatory authority stated that—from April 2017—zolpidem prescription must be performed on a secured prescription pad. This national study aims to evaluate the perception of general practitioners (GPs) towards this new regulation and its link with prescription strategies. Methods: We conducted structured interviews of GPs. Data were collected about GPs’ perception of the measure and therapeutic strategies towards zolpidem. The primary outcome was the description of the GPs’ strategy of prescription, based on the perception towards the new regulation for zolpidem. Results: For 206 GPs, the new regulation was mainly perceived as helpful (61%) and as a difficulty (55%). Other perceptions were the awareness of the risks of zolpidem (18%), awareness of the risks of hypnotics (13%), and nothing changed (5%). Four clusters of GPs were identified. In the clusters with the perception as a difficulty (only or associated with helpful), the GPs who applied the strategy “no modification” for >50% of their patients were more frequently compared to awareness and helpful only clusters (60.8%; 42.9%; 20.4%; 26.7%) (*p* < 0.001). Conclusions: We highlighted an association between the perception of the new regulation of zolpidem prescription by GPs and a strategy of prescription.

## 1. Introduction

Benzodiazepines and Z-Drugs (BZD/Z) are widely prescribed worldwide for anxiety and sleep disorders. Among BZD/Z, zolpidem—a short half-life hypnotic—is one of the most commonly used [1]. Contrary to what was initially supposed in the early phase of its marketed authorization, zolpidem consumption has become associated with abuse and dependence [2,3]. Similar to other BZD/Z, zolpidem is associated with chronic use [4]. In France, the evaluation of the addictive potential of drugs and surveillance of drug dependence is assured by a network of 13 centers for the evaluation of and provision of information on drug dependence (Centres d’Evaluation et d’Information sur la Pharmacodépendance-Addictovigilance (CEIP-A)) [5]. The Nantes addictovigilance center is responsible for monitoring zolpidem. For several years, the number of notifications for substance use disorder (formerly abuse and dependence) related to zolpidem has increased [2,6,7,8]. Two distinct profiles tend to be shown: one related to the hypnotic effects in relation to chronic sleep disorders and another profile related to younger patients searching for the paradoxical amphetamine-like stimulant effects of zolpidem [2]. Specific pharmacoepidemiologic tools, such as the surveillance of falsified prescriptions, have revealed that zolpidem was also among the top listed falsified prescriptions [6].

To limit these phenomena, health authorities and agencies have provided different measures and regulations worldwide. The World Health Organization and the Food and Drug Administration provided alerts regarding the risks associated with zolpidem, especially drug dependence, and suggested dose reduction [9,10]. In France, zolpidem prescription is restricted to the treatment of transient sleep disorders and is limited to 4 weeks, which includes a tapering period (versus 12 weeks for anxiolytic BZD) [11]. Following the emergence of evidence of zolpidem abuse [2] in 2002, a warning regarding the risk of addiction was added to the Summary of Product Characteristics (SPC) of zolpidem. Unfortunately, a new survey in 2011 showed that zolpidem abuse and dependence were increased in the same two subgroups of problematic consumers [3]. Thus, to “limit the risk of abuse and misuse” and “to encourage correct usage”, the French national drug regulatory authority stated that from April 2017 onward, zolpidem prescription must be performed on a secured prescription pad [12].

The main purpose of the ZORRO (ZOlpidem and the Reinforcement of the Regulation of prescription Orders) study, conducted by the CEIP-A of Nantes, was to evaluate the overall impact of the implementation of this regulatory framework for zolpidem prescriptions [13]. Initial results showed a decrease in the prevalence of zolpidem users in the general population [14]. Looking at individual treatment trajectories in long-term users, we identified different clusters: zolpidem continuation, zolpidem discontinuation without replacement with another sedative, and zolpidem replacement with zopiclone or another hypnotic BZD [15]. These clusters were associated with the individual characteristics of zolpidem users.

Beyond the individual characteristics of patients, we can hypothesize that the perception of General Practitioners (GPs) of this new regulation has an influence on the prescription of zolpidem. It is already known that GPs do not always adhere to guidelines [16]. Meanwhile, relationships between perceptions and their links with decisions and patterns of prescription by physicians after new regulations have been implemented, have been poorly investigated in the literature. In a systematic review and meta-analysis conducted in 2018 that measured the impact of medicine regulatory interventions, Goedecke et al., found only 27 studies (less than a fifth of the literature retained) assessing knowledge, behavior or clinical practice; moreover, no study focused on hypnotics [17]. It has been previously demonstrated that GPs in French-speaking regions of Europe had a variable uptake of common preventive recommendations. In a study by Sebo et al., in France and Switzerland, a diversity of attitudes towards regulations was associated with misunderstandings of the current guidelines, barriers to guideline uptake or the absence of agreement between the various recommendations [18].

In this context, the aim of the present study—which is part of the ZORRO study—is to evaluate the perception of GPs towards the new 2017 regulation regarding zolpidem prescription and to investigate the association between perceptions and prescription attitudes after the implementation of the new regulation.

## 2. Materials and Methods

### 2.1. Study Oversight

This study is part of the multimodal ZORRO study. ZORRO is a national study conducted by the CEIP-A in the Nantes University Hospital, aiming to evaluate the overall impact of zolpidem regulatory change on the prescription of hypnotic/sedative and anxiolytic medications. The whole trial included data from physicians, patients and users and took place from 25 July 2018 to 28 January 2020 for the final data collection date for primary outcome measure. The ZORRO study combines (i) an epidemiological perspective with an analysis of the French national health claims database [14,15] and (ii) a clinical perspective with investigations of 449 participants: 206 general practitioners (GPs) and 243 problematic consumers of zolpidem (patients of GPs’ offices or users of specialized centers of drug addiction). The present study is from the clinical perspective and evaluated the perception of the new regulation of zolpidem prescription among GPs. Results from the clinical evaluation of problematic consumers will be presented in another publication.

The study was funded by a grant from the ANSM and was monitored by a multidisciplinary steering committee with pharmacologists, psychiatrists specialized in addiction, pharmacoepidemiologists and general practitioners. The Committee for the Protection of the Population and the Committee (CPP) of expertise in research, studies and evaluations in the field of health approved this study (CPP favorable advice number: 2018-A01070-55 from 18 May 2018). The study was registered at www.clinicaltrial.gov under the reference NCT03584542. The study protocol is available as an open publication [13].

### 2.2. Study Population

The GPs contacted were randomly selected by the drawing of lots from the French health care professionals’ directory or were members of a network of habitual partners of the investigator service. To be included in this ancillary study, GPs must have had a liberal practice of outpatient care and engaged in medical consultations during the time of shifts in the regulation. Oral consent was collected during the inclusion process.

### 2.3. Study Procedures

Identified GPs were called by phone for a short questionnaire. The telephone interviews took place between 25 July 2018 and 5 November 2019. An ad hoc questionnaire was built by a multidisciplinary committee and the questionnaire was tested beforehand on a sample of physicians in order to ensure its acceptability and feasibility. Data were collected regarding (i) GP characteristics, such as duration of activity and city of practice; (ii) the perception of the measure by GPs; and (iii) the therapeutic strategy towards zolpidem prescription.

For the assessment of perceptions related to the new regulation, GPs were asked, “Which of the following expressions best represent your state of mind when you took notice of the regulatory change?”; one or more answers were possible:

- Nothing changed;
- A supplementary difficulty (writing on secured prescription pads);
- Awareness of risks of zolpidem;
- Awareness of risks of hypnotics;
- Helpful for zolpidem discontinuation.

The GPs were asked about the estimated proportion of their patients concerned by each of the following strategies: *no modification*, *zolpidem dosage reduction*, *zolpidem discontinuation without replacement*, and *zolpidem discontinuation with replacement* (initiation of another treatment). GPs also reported some patient characteristics (such as age, sex, duration of the prescription, etc.) that could be related to the choice in the different therapeutic strategies.

### 2.4. Outcomes

The primary outcome was the description of the GPs’ strategy of prescription based on the perception towards the new regulatory framework for zolpidem prescription.

### 2.5. Statistical Analysis

The descriptive analysis of the population of GPs is presented using counts and percentages for categorical variables. The proportions of patients concerned by each strategy of prescription are described using means and 95% confidence intervals (obtained by bootstrapping), as it was a non-normal distribution for the total population and stratified by the different answers regarding perceptions.

GP duration of practice was categorized as follows: <5 years; 6–10 years; 11–20 years; 21–30 years; and >30 years. Based on the size of the city of practice (< or >2000 inhabitants), GPs were categorized as active in rural or urban areas.

GPs were categorized based on their pattern of answers regarding their perceptions of the new regulation, to obtain different groups for subsequent comparisons.

We used Fisher’s test for the comparison of the categorical variable of GP duration of practice and chi2 tests for the comparison of the GP area of practice and the proportion of GPs who had applied the no modification strategy with more than half of their patients. The threshold of statistical significance was fixed at *p* < 0.05. All analyses were conducted using R software version 3.6.1 (R Core Team, Vienna, Austria).

## 3. Results

Of the 1154 GPs selected during the study duration: 485 were contacted, 538 were not contacted despite attempts and 131 were not eligible for the study. Two hundred and six GPs completed the questionnaire, 279 refused to answer (participation rate: 42%).

### 3.1. Description of the Population and Perceptions of the New Regulation

A detailed description of the included population is presented in Table 1. The most represented duration of practice for GPs was over 30 years (30%), followed by 21–30 years (24%) and the three other categories under 20 years (from 12 to 17%). Additionally, GPs mainly had urban practices (89%). Regarding the item on perception, the new regulatory framework for zolpidem was mainly perceived as helpful for discontinuation (61%) and as a difficulty (55%). Other perceptions were present for below a fifth of the population of GPs: awareness of the risks of zolpidem, awareness of the risks of hypnotics, and nothing changed (18%, 13% and 5%, respectively).

### 3.2. Description of the Different Strategies Applied with Regard to the New Regulation

**Figure 1:**
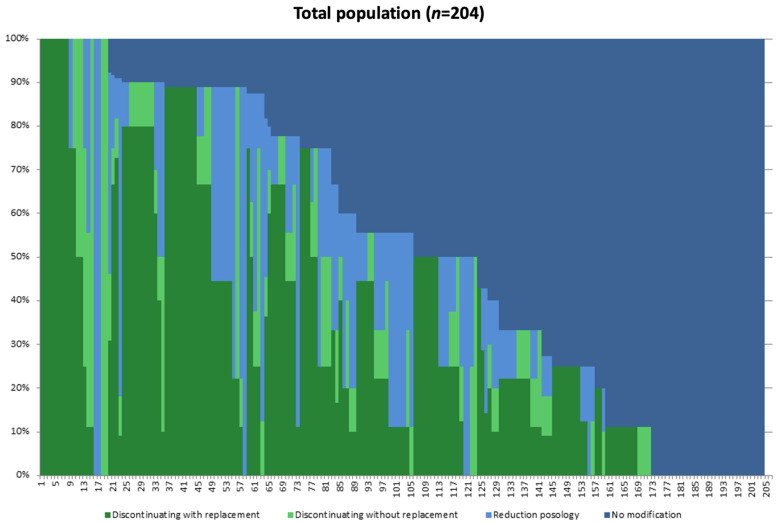
Percentages of population of patients concerned by each strategy for every GP. Legend: GPs are represented individually on x-axis (one vertical line represents one GP) and proportion of patients declared by GPs (as percentages) concerned by each strategy are represented on y-axis. For example, GP_37_ had applied discontinuation with replacement strategy for 80% of his patients, whereas the no modification strategy has been applied for 20% of this GP patients.

A graphical representation of the percentage of patients concerned by each strategy for each of the 204 GPs is provided in Figure 1. We observed that the strategy *no modification* concerned about half of all the individual strategic decisions applied by the GPs, followed by *discontinuation of zolpidem with replacement*, *reduction in zolpidem dosage* and, finally, *discontinuation of zolpidem without replacement*. The *no modification* strategy was the only strategy employed by 32/204 GPs (16%), and 81/204 (40%) applied this strategy with more than 50% of their patients. The mean percentage of the patients to which the *no modification* strategy was applied was 49% (IC_95_: 11–75); this was followed by the *discontinuation of zolpidem with replacement* strategy in 33% of patients (IC_95_: 28–38) and *reduction in zolpidem dosage* then *discontinuation of zolpidem without replacement* strategies in 13% (IC_95_: 10–17) and 5% (IC_95_: 3–8) of patients, respectively.

### 3.3. Different Strategies Applied towards Zolpidem Prescription Based on Perceptions towards the New Regulation

Of the 205 GPs who responded to the perception item (1 missing data), these were divided into four main Groups based on the patterns of responses to the perception item. Three patterns of answers were evident: (i) helpful for discontinuation; (ii) difficulty; and (iii) helpful for discontinuation and difficulty. For the fourth pattern, a common denominator was the presence of at least one answer about the awareness of the risks of zolpidem and/or hypnotics. Thus, the Group awareness of the risks of zolpidem and/or hypnotics is composed of 21 GPs who answered awareness of risks of zolpidem and/or hypnotics and difficulty; 18 GPs who answered awareness of risks of zolpidem and/or hypnotics and helpful for discontinuation; and 6 GPs who answered awareness of risks of zolpidem and/or hypnotics (only). Among the unclassified GPs, 11 stated that the new regulation had not changed anything, and 2 provided a pattern of answers that was unclassifiable. The final Groups were as follows:

- Helpful for discontinuation (only), *n* = 54.
- Difficulty (only), *n* = 51.
- Awareness of risks of zolpidem and/or hypnotics (and potentially other answers), *n* = 45.
- Helpful for discontinuation and difficulty, *n* = 42.

**Figure 2:**
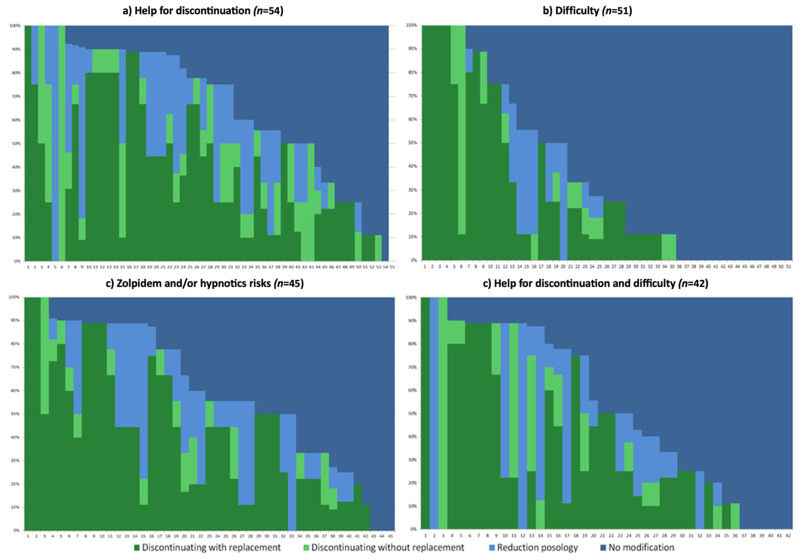
Percentages of population concerned by each strategy according to GPs’ answers to perception item. Legend: GPs are represented individually on x-axis (one vertical line represents one GP) and proportion of patients declared by GPs (are percentage) concerned by each strategy are represented on y-axis. For example, GP_3_ of the Help for discontinuation group had applied the discontinuation with replacement strategy for 50% of his patients, where the discontinuation without replacement strategy had been applied for 50% of this GP patients.

Detailed results of the Group comparisons are presented in Table 2, and the individual percentages of patients concerned by each strategy, stratified by GP perceptions, are represented in Figure 2.

In the helpful for discontinuation (only) Group, GPs mainly had less than 5 years of practice (30%); other durations of practice were between 13% and 20%, and 87% of GPs had urban practices. In this Group, two GPs applied one strategy for 100% of their patients: *no modification* (*n* = 1) and *discontinuation without replacement* (*n* = 1). They were a fifth of the GPs who applied the *no modification* strategy for >50% of their patients. The main strategy applied was *discontinuation with replacement* for an average of 40% of their patients, followed by the *no modification* strategy for one-third of their patients, followed by *reduction in dosage* and *discontinuation without replacement* strategies.

In the difficulty (only) Group, approximately half of the GPs had over 30 years of practice, and other durations of practice were 29% for 21–30 years of practice and between 4 and 14% for categories under 20 years of practice. They were 88% of the GPs with an urban practice. In this Group, 16/51 GPs had applied the *no modification* strategy, and 6 had applied the *discontinuation of zolpidem with replacement* strategy for 100% of their patients. There were 61% of the GPs who applied the *no modification* strategy for >50% of their patients. The main strategy applied was the *no modification* strategy for two-thirds of their patients, followed by *discontinuation with replacement* for approximately a quarter of the patients. The two other strategies were applied to 5% or less of their patients.

In the awareness of risks of zolpidem and/or hypnotics Group, 38% of the GPs had over 30 years of practice, and other durations of practice were between 13% and 22%; 93% of GPs had an urban practice. In this Group, 3/45 GPs had applied the *no modification* strategy, and 2 had applied the *discontinuation of zolpidem with replacement* strategy with 100% of their patients. More than a quarter of these GPs applied the *no modification strategy* with >50% of their patients. The two main strategies applied were *discontinuation with replacement* and *no modification,* with more than 40% of their patients for each, followed by *reduction in dosage* and *discontinuation without replacement* strategies.

In the helpful for discontinuation and difficulty Group, GPs mainly (29%) had between 21 and 30 years of practice, and other durations of practice were between 14% and 21%; most GPs had urban practices (88%). In this Group, 6/42 GPs had applied the *no modification* strategy with 100% of their patients, and 1 applied each of the other strategies for 100% of their patients. Overall, 43% applied the *no modification* strategy with >50% of their patients. The main strategy applied was the *no modification* strategy for approximately half of their patients, followed by *discontinuation with replacement* with one-third of their patients and a *reduction in dosage* and *discontinuation without replacement* strategies.

The four Groups of GPs categorized by their perception of the new regulation were significantly different in terms of duration of practice (*p* = 0.012) but not in terms of the area of their practice (*p* = 0.776). The proportion of GPs who had applied the *no modification strategy* for more than half of their patients was significantly different between groups (*p* < 0.001), with a more pronounced difference between the difficulty (only) and the helpful for discontinuation (only) Groups: 61% versus 20%, respectively.

The duration of prescription was in all cases the main reason for choosing a particular strategy for a patient according to the GPs. A duration of zolpidem administration of more than 3 months was the reason, with the exception in situations where discontinuation without replacement was the reason of choice in patients with a prescription of no longer than 3 months. An age over 65 years old was the second reason cited for choosing strategies related to zolpidem continuation. Consideration of psychiatric comorbidities was also cited for zolpidem continuation (with or without zolpidem dosage reduction). Gender was always the reason least cited for any particular strategy.

## 4. Discussion

The aim of this study was to evaluate the prescription strategies by GPs since the implementation of a new regulation regarding zolpidem prescription, based on their perception of the regulation. We found that the perception of the new regulation of zolpidem as only a difficulty was associated with a more frequent status quo strategy of the zolpidem prescription, whereas the perception involving help for discontinuation was more closely associated with discontinuing zolpidem administration (with or without replacement) and reducing the dosage. The awareness of the risks of zolpidem and/or hypnotics patterns were equally shared between the strategies of *no modification* or *discontinuation of zolpidem with replacement*. Those with the more balanced pattern (help for discontinuation and difficulty) were more likely to continue with the same prescription. We also highlighted some significant differences between the different GP Groups based on their perceptions, notably on the duration of practice, with more experienced GPs having perception patterns that included awareness of risks and difficulty. To our knowledge, this is the first study to address the question of the impact of a new regulation on perception and its association with the prescription strategy adopted by GPs. Additionally, ZORRO is the first study addressing a before/after comparison regarding the zolpidem issue.

Considering the overall results, more than half of the GPs declared that the new regulation for zolpidem prescription is perceived as a difficulty (55%) for their practice but also helpful for discontinuation (61%), which can appear contradictory. This contradiction was also found when considering the high rate of the perception helpful for discontinuation with the high prevalence of a *no modification* strategy. However, when looking at the results more closely and across the Groups based on their perceptions, we saw that the proportion of GPs who applied the *no modification* strategy with more than the half of their patients was significantly less represented in the helpful for discontinuation and the awareness of risks of zolpidem and/or hypnotics Groups than in the perception of difficulty (only or with help for discontinuation) Group. This trend emphasizes that the perception of the new regulation as a difficulty only is a barrier to modifications designed to discontinue zolpidem prescription. The characteristics of the GPs in those Groups are also different; notably, the GPs that had the perception that the regulation was helpful for discontinuation only were more frequently practicing for less than 5 years (we can assume these were younger GPs), whereas those who perceived the regulation as a difficulty only were more frequently practicing for over 30 years. This indicated that more experienced GPs had less willingness to adapt their prescription strategies to guidelines and regulations, which should be considered in health politics given that physicians’ mean age in France is more than 50 years old [19].

The results presented here are in concordance with the initial results of the ZORRO study [14,15] using a database of reimbursement claims of drugs, showing a decrease in French zolpidem consumers from 2.79% to 1.48%, with a fourfold increase in zolpidem discontinuation. With this database approach, different clusters of drug delivery after the new regulation of zolpidem prescription were also identified: the main proportion of patients (42%) continued zolpidem, which is consistent with the main strategy of *no modification* of zolpidem prescription found in our study. However, the other magnitudes were different: *zolpidem discontinuation without replacement* was the second most frequent cluster in the database (31%), whereas it was a less frequent attitude expressed in our sample, and discontinuation with replacement (zopiclone or other BZDs) was the second most frequent attitude in our sample but not in the database approach (27%). This discrepancy may reflect divergent perceptions between GPs and patients; when the GPs thought to substitute zolpidem with another drug, patients seized the opportunity to stop hypnotic administration. Another possible explanation for this discrepancy is the different observation periods between the database and clinical approaches that can result in an initial replacement of zolpidem before a permanent stop of hypnotic administration.

As the zolpidem issue has been a major health concern for decades, an essential question is how health politics can guide perceptions towards a new regulation. Previous studies in the analgesic domain showed that significant prescribing changes occurred when national advice and guidelines were issued [20]. The perception of a regulation by French GPs as a difficulty is not novel. In a study by Daveluy et al. [21] regarding the generalization of tamper-resistant prescriptions for narcotics, more than 60% of GPs were against the regulation and made arguments based on the excessive administrative burdens, technical problems of software and/or printers, and stigmatization of psychiatric illnesses. A few GPs perceived the new regulation as an awareness of the risks of zolpidem and/or other hypnotics– but were the others already aware or not about those risks? Nevertheless, this finding is in line with previous results in British and German GPs, where Z-Drugs were perceived as safer than BZDs [22,23]. It has been previously shown that the impact of a regulation was dependent on its delivery: national communication from regulation authorities to physicians (as in the previous case of the warning on valvulopathy risk associated with pergolide use) [24] seemed underperforming, whereas regional communication and/or media delivery (as in the case of the SNAID, celecoxib) of the regulation was more likely to be effective in terms of prescription behavior [24,25]. In this study, we showed that beyond the communication regarding the new national regulation of zolpidem prescription (considered similar for every GP), characteristics that can be linked to perceptions of the regulation can influence prescribing behaviors after the regulation. Astonishingly, the perception of danger was more present in the more “experienced” Group of GPs, corresponding to a pattern that was less prone to change their prescription strategies. However, this failure to change pattern among GPs was also associated with viewing the regulation as a difficulty. In contrast, perception of the regulation as a helpful measure was associated with less conservative strategies. Thus, for subsequent health policies regarding drug prescription behavior, particular emphasis must be placed on supporting prescribers in modifying their habits so that they can perceive the interests of patients. The heterogeneity in GP patterns suggests that tailored means that correspond to the different profiles are needed. Incentives and individual measures should also be offered to groups of GPs identified as less likely to modify their prescribing habits. These approaches to implementing a communication plan and individual measures of new regulations could work synergistically.

Considering patient characteristics, the main reason (regardless of the strategy employed) for choosing a particular prescription strategy was the duration of prescription. We noticed a gap between recommendations and practice, as BZD/Z administration should not exceed one or three months of prescription, but a duration over three months was the main reason for not changing the prescription. Moreover, for the *no modification* and *zolpidem dosage reduction* strategies, an age over 65 years old was the second reason for zolpidem continuation. Age has already been identified in the literature as a factor associated with BZD/Z prescription renewal [16,26]. Here, too, the patterns appear contradictory given that older subjects are more prone to side effects and risks of long-term zolpidem prescription [27,28,29]. These elements highlight the difficulty for GPs to manage sleep disorders, balanced between a desire to help patients with widespread symptoms that can have immediate and major repercussions on quality of life and the need for reductions in prescriptions to avoid side effects and drug dependence [30,31,32,33,34]. In their study collecting two dozen (12 hospital doctors and 12 GPs) semi-structured interviews about hypnotic prescriptions, Weiß et al. [35] described contextual factors, such as patient demands and time resources, for prescribing hypnotics against an attempt to act rationally. Comparing GPs and hospital doctors in this study, they found that physicians could feel “pressured” to prescribe, and GPs were in “conflict” regarding prescribing “something to sleep” for their patients. The presence of psychiatric comorbidities has also been cited as a factor for choosing “conservative” strategies (no modification or a switch for another medication). Sleep disorders are common symptoms of psychiatric disorders such as mood disorders or anxiety disorders [36]. Meanwhile, the presence of psychiatric disorders often needs curative treatment (as an antidepressant for major depressive disorder or generalized anxiety disorder) for patients, and hypnotics must be prescribed for a short period [37].

The originality of our work lies in the investigation of GPs’ perception of the new framework of zolpidem prescription, its link with the strategy of prescription employed and its complementarity with the database results of the previously published ZORRO study. Our sample is nationwide and has various profiles of GPs that reflect a diversity of practices. The number of completed questionnaires that were obtained allowed us to perform statistical analysis, although the response rate of below one-fifth of the selected GPs could appear low. On the other hand, our study has several limitations. First, the results were self-reported and do not guarantee that what was declared was what has been done. Furthermore, answers could be subject to self-report bias and reflect an “ideal” point of view of the GPs. Furthermore, the telephone interview took place between the second quarter of 2018 and the end of 2019, which may have been more than two years after the announcement and implementation of the zolpidem regulation. This raises the concern of possible memory bias about how GPs perceived the regulation and what they have done regarding zolpidem prescriptions. Additionally, less than one-fifth of the GPs selected completed the whole questionnaire raising non-response bias.

## 5. Conclusions

In this study, we investigated and related the perception of a new regulation with the prescription strategy. We highlighted a GP profile involving less clinical experience and a perception of the change in regulation being helpful, for whom a new therapeutic strategy can be conceivable. At the same time, there is concern about the difficult management of sleep disorders and being sensitive to patients’ particularities. Beyond national regulation, tailored measures aimed at GPs’ perceptions are needed to ensure their success.

## Figures and Tables

**Table 1:** Characteristics of respondents and perception toward the new regulation framework for zolpidem.

Duration of Practice		*n* = 204
	<5 years	34 (16.7%)
	6–10 years	24 (11.8%)
	11–20 years	35 (17.2%)
	21–30 years	49 (24.0%)
	>30 years	62 (30.4%)
Location of practice		*n* = 206
	Urban	183 (88.8%)
	Rural	23 (11.2%)
Perception		*n* = 205
	Difficulty	114 (55.3%)
	Awareness of risks of zolpidem	36 (17.5%)
	Awareness of risks of hypnotics	26 (12.6%)
	Helpful for discontinuation	125 (60.7%)
	Nothing changed	11 (5.4%)

**Table 2:** Different proportions of patients concerned by each strategy and GPs’ characteristics according to their pattern of perception of the new regulation.

	Helpful for Discontinuation Only	Difficulty Only	Awareness of Risks of Zolpidem and/or Hypnotics	Helpful for Discontinuation and Difficulty	
	*n* = 54	*n* = 51	*n* = 45	*n* = 42	
Strategy employed		Proportion of patients concerned, mean [IC_95_]			
Discontinuation with replacement	39% (30–49)	24% (15–35)	42% (31–53)	31% (21–43)	
Discontinuation without replacement	10% (5–15)	3% (0.5–10)	2% (0–6)	8% (3–17)	
Reduction posology	19% (13–27)	5% (2–10)	14% (8–22)	14% (7–26)	
No modification	33% (24–42)	68% (56–78)	42% (31–53)	47% (35–58)	
	GPs who have applied this strategy for >50% of their patients, number (%)				*p*-value
No modification	11 (20.4%)	31 (60.8%)	12 (26.7%)	18 (42.9%)	<0.001
		GPs’ characteristics, number (%)			
Duration of practice					*p*-value
<5 years	16 (29.6%)	3 (5.9%)	7 (15.6%)	6 (14.3%)	
6–10 years	7 (13.0%)	2 (3.9%)	5 (11.1%)	9 (21.4%)	
11–20 years	10 (18.5%)	7 (13.7%)	6 (13.3%)	8 (19.0%)	0.01
21–30 years	11 (20.4%)	15 (29.4%)	10 (22.2%)	12 (28.6%)	
>30 years	10 (18.5%)	24 (47.1%)	17 (37.8%)	7 (16.7%)	
Area of practice					
Urban	48 (87.3%)	45 (88.2%)	42 (93.3%)	37 (88.1%)	0.776

Legend: IC_95_ = 95% confidence interval.

## Data Availability

Given the confidentiality of the data, our ethics committee (GNEDS) is preventing us from making our data set publicly available. However, we are willing to make our data available upon request as we consider that it is important for open and reproducible science, and thus we will ensure that all interested and qualified researchers will be able to be granted access.
